# *In silico* and *in vitro* metabolism of ribociclib: a mass spectrometric approach to bioactivation pathway elucidation and metabolite profiling

**DOI:** 10.1039/d0ra01624a

**Published:** 2020-06-12

**Authors:** Thamer A. Alsubi, Mohamed W. Attwa, Ahmed H. Bakheit, Hany W. Darwish, Hatem A. Abuelizz, Adnan A. Kadi

**Affiliations:** Department of Pharmaceutical Chemistry, College of Pharmacy, King Saud University P.O. Box 2457 Riyadh 11451 Saudi Arabia hdarwish@ksu.edu.s +966 1146 76 220 +966 1146 70237; Students' University Hospital, Mansoura University Mansoura 35516 Egypt; Analytical Chemistry Department, Faculty of Pharmacy, Cairo University Kasr El-Aini St. Cairo 11562 Egypt

## Abstract

Ribociclib (RBC, Kisqali®) is a highly selective CDK4/6 inhibitor that has been approved for breast cancer therapy. Initially, prediction of susceptible sites of metabolism and reactivity pathways were performed by the StarDrop WhichP450™ module and the Xenosite web predictor tool, respectively. Later, *in vitro* metabolites and adducts of RBC were characterized from rat liver microsomes using LC-MS/MS. Subsequently, *in silico* data was used as a guide for the *in vitro* work. Finally, *in silico* toxicity assessment of RBC metabolites was carried out using DEREK software and structural modification was proposed to reduce their side effects and to validate the bioactivation pathway theory using the StarDrop DEREK module. *In vitro* phase I metabolic profiling of RBC was performed utilizing rat liver microsomes (RLMs). Generation of reactive metabolites was investigated using potassium cyanide (KCN) as a trapping nucleophile for the transient and reactive iminium intermediates to form a stable cyano adduct that can be identified and characterized using mass spectrometry. Nine phase I metabolites and one cyano adduct of RBC were characterized. The proposed metabolic pathways involved in generation of these metabolites are hydroxylation, oxidation and reduction. The reactive intermediate generation mechanism of RBC may provide an explanation of its adverse reactions. Aryl piperazine is considered a structural alert for toxicity as proposed by the DEREK report. We propose that the generation of only one reactive metabolite of RBC in a very small concentration is due to the decreased reactivity of the piperazine ring compared to previous reports of similar drugs. Docking analysis was performed for RBC and its proposed derivatives at the active site of the human CDK6 enzyme. Methyl-RBC exhibited the best ADMET and docking analysis and fewer side effects compared to RBC and fluoro-RBC. Further drug discovery studies can be conducted taking into account this concept allowing the development of new drugs with enhanced safety profiles that were confirmed by using StarDrop software. To the best of our knowledge, this is the first literature report of RBC*in vitro* metabolic profiling and structural characterization and toxicological properties of the generated metabolites.

## Abstract 2

Nine phase I metabolites and one product of KCN trapping of RBC were characterized. Aryl piperazine is considered a structural alert for toxicity as proposed by the DEREK report. Methyl-RBC exhibited less toxicity and more binding affinity to CDK6.

## Graphical abstract

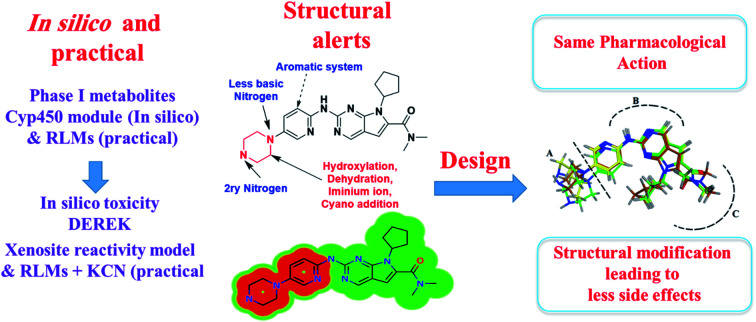

## 1 Introduction

A malignant tumor consists of cancer cells that are characterized by uncontrolled multiplication and the potential to metastasize.^1^ Worldwide, breast cancer is considered the prevalent diagnosed cancer in women as it affects around 12% of women globally.^2^ Breast cancer in Saudi Arabia occurs at an estimated 22% prevalence among new cancer diagnosis in women.^3^ There are a group of targeted drugs that precisely affect gene modifications in cancer cells that aid in stopping the cells from growing or invading. Tyrosine kinases (TK) are crucial targets owing to their significant part in the modulation of growth factor signaling.^4^ Regulating the activity of TK in the cell controls various essential processes such as proliferation, cell death and cell cycle.^5^

Cyclin-dependent kinase 4 and 6 (CDK4/6) as a class of TKI play an important part in cell propagation. Uncontrolling CDK4/6 pathway affected the biology of breast cancer.^6^ Recently, there are some development in the selective CDK4/6 inhibitors, which give an acceptable efficacy and manageable safety results. Three CDK4/6 inhibitors have been FDA approved for breast cancer: Ribociclib (RBC, Kisqali®), Palbociclib (Ibrance®) and Abemaciclib (Verzenio®).^7^

**Fig. 1:**
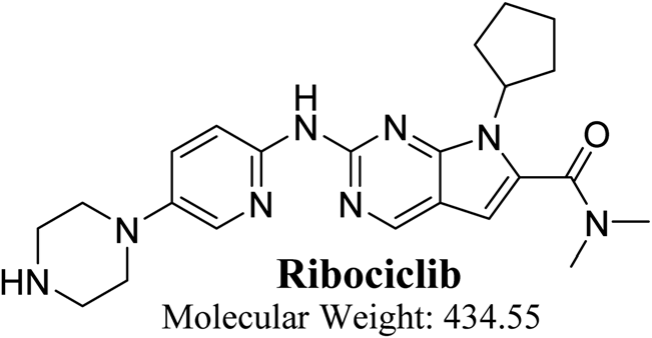
Chemical structure of ribociclib.

RBC (7-cyclopentyl-*N*,*N*-dimethyl-2-{[5-(1-piperazinyl)-2-pyridinyl]amino}-7*H*-pyrrolo[2,3-*d*] pyrimidine-6-carboxamide) is a selective inhibitor of CDK4/6 that demonstrated antitumor effect in preclinical and clinical studies (Fig. 1). On 13 March 2017, FDA approved RBC in addition to an aromatase inhibitor for the cure of postmenopausal women with human epidermal growth factor receptor 2 negative metastatic, hormone receptor positive or advanced breast cancer. The most common adverse reactions of RBC (20% patients) were neutropenia, leukopenia, fatigue, alopecia, headache and back pain. Gastrointestinal disorders were also common as diarrhea, nausea, constipation and vomiting.^8^RBC also causes QT interval prolongation (7.5% patients) in a concentration reliance manner. Nevertheless, these adverse effects were remarkable and might be serious especially with long term use.^7^

RBC contains piperazine ring moiety that is considered a structural alert for toxicity. Our research group previously studied reactive metabolites formations for abemaciclib that contains piperazine group also in its chemical structure.^9^ The current work proves that RBC formed one reactive intermediates through a specific bioactivation pathway. KCN was utilized as a capturing tool to trap the generated reactive intermediates and the bioactivation mechanism were supposed.^10–15^ Hydroxylation at a carbon alpha to tertiary nitrogen atom of piperazine ring is known to generate iminium ions, that are considered hard electrophiles and can be trapped by cyanide anions (nucleophile) to forming cyano conjugate. Reactive intermediate formation is a probable explanation for RBC toxicity.^16–19^ Iminium intermediates initiate several toxic side effects through binding covalently to a DNA base.^13,20–22^ Furthermore, *in silico* study was done for susceptible metabolic site prediction at RBC chemical structure.

In the current work, we found that the ability of the piperazine ring in RBC to form reactive intermediates is less compared to abemaciclib and palbociclib,^9,23^ this is believed to be due to the decreased basicity resulting from being attached to the electron withdrawing pyrimidine ring, thus less chance to form iminium intermediates. As a result, RBC only generates one reactive metabolite in a very small concentration and therefore is proposed to possess less toxicity as was confirmed using *in silico* and *in vitro* work. This could be a new strategy for reducing the side effects of new developed drugs without affecting its pharmacological activity that was proposed using StarDrop software. Knowing the bioactive center and structural alerts in RBC structure helped in making targeted modifications to improve its safety and retain its efficacy that was confirmed using DEREK software. Nine *in vitro* phase I metabolites and one cyano conjugate of RBC were characterized. The proposed pathways involved oxidation, reduction, cyanide addition and hydroxylation. *In silico* toxicity assessment of RBC metabolites was carried out using DEREK software and structural modification were proposed to reduce their side effects and to validate the bioactivation pathway theory using StarDrop software. The docking studies were carried out for RBC and predicted derivatives using the human CDK6 complex obtained from Protein Data Bank (PDB code 5L2T, https://www.rcsb.org/structure/5L2T).^24^ ADMET descriptors (absorption, distribution, metabolism, excretion and toxicity) of the RBC and proposed derivatives were determined using Discovery Studio 4.5 client (Accelrys, San Diego, CA, USA). To the best of the authors' knowledge, no previous study on the *in silico* or *in vitro* metabolism of RBC has been reported.

## 2 Chemicals and methods

### 2.1. Chemicals

All chemicals and solvents are analytical grade. Sprague Dawley rats were used for RLMs preparation.^21,25–29^RBC was procured from MedChem Express company (NJ, USA). Ammonium formate, acetonitrile, formic acid and potassium cyanide were procured from Sigma-Aldrich company (USA). Sprague-Dawley rats were taken from experimental animal care center (King Saud University). All animal experiments were performed following the standards set forth in the experimental Animal Use and Care Guidelines of the National Institutes of Health and the Supervision of Animal Experiments Committee. The study was validated and approved by the Committee for Animal Ethics of the Pharmacology Department at King Saud University (no. KSU-SE-19-76).

### 2.2. Chromatographic conditions

An Agilent 6410 triple quadrupole fitted to an electrospray ionizer (ESI) with an Agilent 1200 rapid resolution liquid chromatographer (RRLC) was utilized. The most optimized chromatographic and mass parameters were selected for RBC and its related metabolites. Fragment ions for RBC and their metabolites were formed inside the second hexapole (collision cell) by collision induced dissociation technique using high purity nitrogen. The chosen chromatographic conditions for resolution of RBC metabolites are shown in Table 1.

**Table 1:** Liquid chromatography and mass spectrometry chosen parameters

Mobile phase	Binary system of 0.1% formic acid in H_2_O (A) and ACN (B)		ESI source	Positive ESI
	0.25 mL min^−1^			High purity N_2_ gas drying gas at 12 L min^−1^ with pressure (60 psi)
	Elution time: 65 min			ESI temperature: 350 °C
				Capillary voltage: 4000 V
Agilent zorbax eclipse plus C_18_ column	Length	150 mm	Modes	Mass scan and fragment ion
	ID	2.1 mm	Collision gas	High purity N_2_
	Particle size	3.5 μm	Analytes	RBC and its reactive metabolites
	T	22 ± 1 °C	Mass parameters	Fragmentor voltage (FV): 135 V
Gradient elution system	Time in min	% ACN		Collision energy (CE): 20 eV
	0	5		
	5	5		
	40	30		
	60	90		
	65	5		

### 2.3. *In silico* prediction of RBC metabolites using WhichP450™ metabolism module of StarDrop software

Our objective is to identify the vulnerability of key sites of metabolism, as revealed by decreasing the site lability that indicated by the composite site lability (CSL) and also the predicted regioselectivity of metabolism by the major isoforms predicted to be responsible for metabolism The result from the WhichP450™ module, shown by the pie chart used for indication of most likely cyp450 isoform that has a major role in RBC metabolism.

### 2.4. *In silico* prediction of RBC reactive metabolites using XenoSite reactivity model and DEREK software

XenoSite reactivity module *in silico* experiments were performed,that are freely available at http://swami.wustl.edu/xenosite, to detect the vulnerable sites reactive intermediates.^30^ This prognostic module is established on neural networks of more than 680 molecules. The software has the advantage of short run time.^31,32^ The chemical structure of RBC (SMILES format) was uploaded to the online website for the XenoSite reactivity module. DEREK software was utilized to screen for structural alerts and to confirm our bioactivation proposal. DEREK software was used also to propose structural modification at the supposed bioactive centres that stop the toxicity sequence.

### 2.5. RLM incubations

RBC was solubilized in dimethyl sulfoxide (DMSO). Protein conc. of the prepared RLMs was determined using Lowery method.^33^RBC (20 μM) was incubated with RLMs (1 mg mL^−1^) in phosphate buffer (50 mM Na/K and 3.3 mM MgCl_2_) at pH 7.4. One mM NADPH was added to initiate the metabolic reaction. One mg mL^−1^ RLMs was used to confirm the absence of non-specific protein binding. One mM KCN was added in the experiments for reactive metabolites detection. The metabolic reactions were done at thermostated shaking water bath (37 °C for 60 min.). Two mL of ACN (ice cold) was added to stop the metabolic reaction by denaturation of enzymes protein. Centrifugation at 9000*g* was done for 10 min at 4 °C to precipitate proteins. The clear supernatants were evaporated under a stream of nitrogen gas then reconstitution in mobile phase. The reconstituted samples were injected into the mass spectrometer system.^10,14,21,34^ Two controls were performed in the absence of RLMs or NADPH to verify that RBC phase I metabolites were metabolically generated. *In silico* toxicity studies of the generated metabolites were performed using DEREK software.

### 2.6. Confirmation for the generation of RBC reactive metabolites

The same RLMs incubations procedure with RBC (Section 2.3) was repeated with 1.0 mM KCN that was added before the addition of NADPH to capture reactive iminium intermediates. This step was performed three times to verify the outcomes. Two controls were performed either in the absence of KCN or NADPH to verify the confirmation of cyano conjugates are generated owing to metabolic bioactivation.

### 2.7. Characterization of RBC reactive intermediates

Extracted ion chromatograms (EIC) and full mass range scan were utilized for characterization and localization of RBC metabolites in the extracts of various incubation mixtures, while generated fragment ions were utilized for reconstructing the chemical structure of RBC related metabolites.

### 2.8. *In silico* ADMET analysis for the proposed RBC derivatives

ADMET descriptors (absorption, distribution, metabolism, excretion and toxicity) of the RBC and proposed derivatives were determined using Discovery Studio 4.5 client (Accelrys, San Diego, CA, USA). ADMET descriptors protocol was used to carry out these studies. These descriptors include human intestinal absorption, solubility of each compound in water at 25 °C, blood–brain penetration (blood brain barrier, BBB) after oral administration, Cytochrome P450 2D6 (CYP2D6) enzyme inhibition, potential liver toxicity, and plasma protein binding.

### 2.9. Molecular docking for the proposed RBC derivatives

Molecular Operating Environment (MOE; Chemical Computing Group Inc., Montreal, Canada) software was used to predict the proposed binding mode, affinity, preferred orientation of RBC and its proposed derivatives (methyl and fluoro) at the active site of the human CDK6 enzyme. The CDK6 enzyme structure coordinates were obtained from Protein Data Bank (https://www.rcsb.org/structure/5L2T:ID:5L2T)^35^ with adequate resolution value (resolution: 2.37 Å). Before the process, the compounds were designed by adding hydrogen atoms after water molecules were extracted over a complete structure. The next step is to add atomic charges, after which the Merck Molecular Force Field (MMFF94x) was adjusted in addition to other essential parameters. The compounds were then transferred into an MDB format for the docking process. The co-crystalline PDB protein format was also set to be ready for docking. To begin with, partial protein charge was optimized with the force field method and an energy refinement was applied to build up an Assisted Model (AMBER 99). After that, polar hydrogen atoms were added, next, the ligand of crystal was selected as domains for docking. The selected sides have finally been docked until they have achieved the most stable interaction after several trials. The energy value for each pose was measured on 30 trials using London dG and GBVI/WSA dG rescoring methods that were twice improved by triangle Matcher methods. On docking complexes various data were collected as; kind of bonds and energy scoring. The H-bonding effectiveness shall be no more than 3.5 Å. The H-bond lengths with amino acids have been used to characterize the interaction performance.

## 3 Results and discussion

### 3.1. Results of *in silico*RBC metabolites prediction

**Fig. 2:**
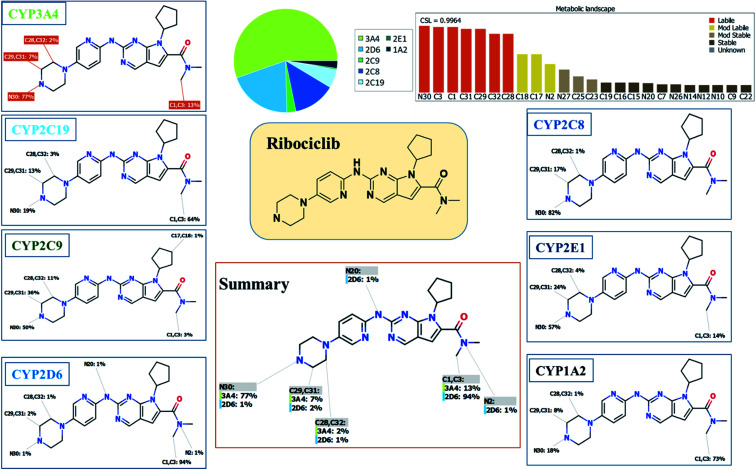
Proposed metabolic sites for RBC by StarDrop WhichP450™ module.

The Metabolic Landscape for RBC indicates the lability of each site with respect to metabolism by CYP3A4 in absolute terms, to guide the prediction of RBC metabolites and also the optimization of chemical structure for improving metabolic stability. This indicates that N30, C31, C29, C32 and C28 on the piperazine, and C1 and C3 of *N*,*N*-dimethyl carboxamide are predicted to be labile that matched with experimental work. The CSL is shown in the top-right of the metabolic landscape, as well as in the P450 column of the data set. The result from the WhichP450™ module, shown by the pie chart used for indication of most likely cyp450 isoform that has a major role in RBC metabolism (Fig. 2). According to the pie chart, Cyp3A4 isoform has the major role in RBC metabolism.

### 3.2. Results of *in silico*RBC bioactivity and toxicity prediction

**Fig. 3:**
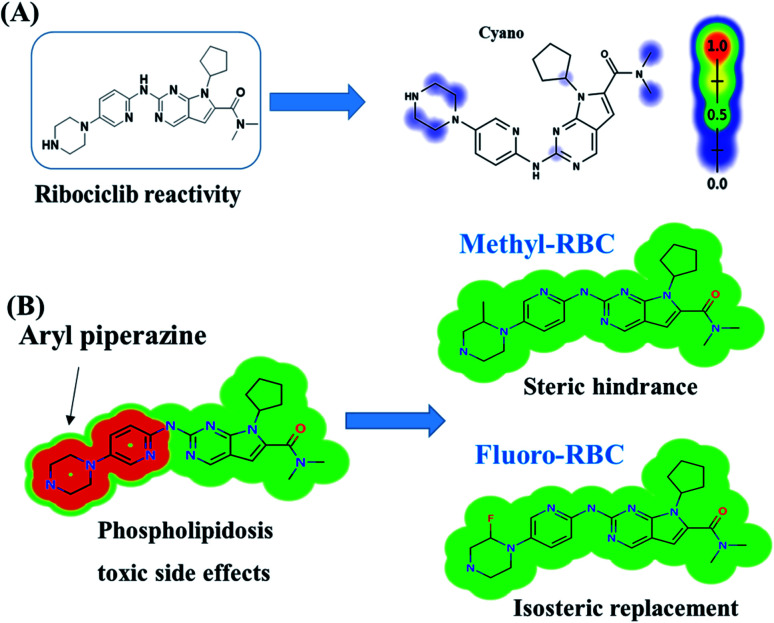
Predicted bioactive sites of RBC by Xenosite web predictor showing cyano bioactive centers and the faint blue color indicates low ability to form bioactive intermediate (A). Structural alert for phospholipidosis of RBC showing the effect of small structural modifications using DEREK software (methyl-RBC and fluoro-RBC) (B).

RBC reactivity was supposed using reactivity module in Xenosite web page as shown in Fig. 3. Consecutively, based on *in silico* predictions and the literature knowledge a list of probable metabolites and reactive intermediates was set. The expected potential atomic sites for the bioactivation and cyanide attack in RBC chemical structure are dimethyl group attached to *N*,*N*-dimethyl carboxamide, cyclopentyl group, pyrimidine and two are α carbon atoms adjacent to the nitrogen atoms of piperazine ring. The faint blue color reveals the less probability to form reactive intermediates that was approved by *in vitro* experiments (Fig. 3A).

*In silico* toxicity assessment of RBC metabolites was carried out using DEREK software and structural modification were proposed to reduce their side effects and to validate the bioactivation pathway theory using StarDrop software (Fig. 3). RBC, M1 and M3 show phospholipidosis due to aryl piperazine moiety. Table 2 shows a complete list of *in vitro*RBC metabolites with DEREK results for proposed toxicity profile. Aryl piperazine (structural alert) was proposed to be the reason for RBC toxicity using DEREK software that matched with our bioactivation theory. Small structural modification at the α carbon to N27 stopped the toxicity that confirmed our proposed theory for RBC toxicity (Fig. 3B).

**Table 2:** Qualitative toxicity prediction of the RBC and its metabolites by DEREK analysis

RBC and its metabolites	Phospholipidosis	Genotoxicity	Organ toxicity	Teratogenicity	Carcinogencity	Skin sensitization	Chromosome damage, genotoxicity and mutagenicity
RBC	✓	NA	NA	NA	NA	NA	NA
M1	✓	NA	NA	NA	NA	NA	NA
M2	✓	NA	NA	NA	NA	NA	NA
M3	NA	NA	NA	NA	NA	NA	NA
M4	NA	NA	NA	NA	NA	NA	NA
M5	NA	NA	NA	NA	NA	NA	NA
M6	NA	NA	NA	NA	NA	NA	NA
M7	NA	NA	NA	NA	NA	NA	NA
M8	NA	NA	NA	NA	NA	NA	NA
M9	NA	NA	NA	NA	NA	NA	NA

### 3.3. Fragment ion study of RBC

**Fig. 4:**
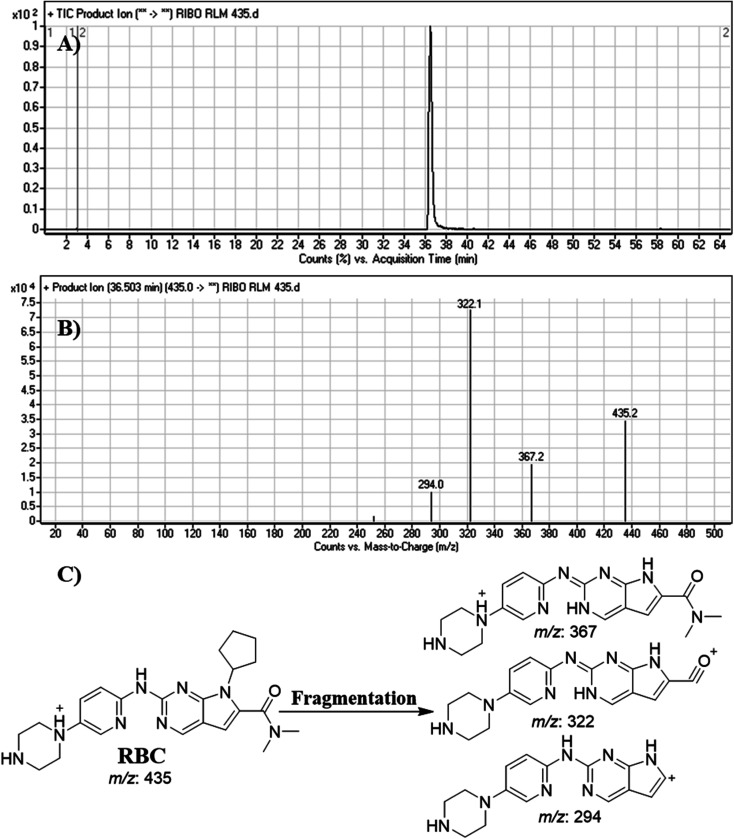
Product ion chromatogram of RBC (A). Product ion mass spectrum of RBC (B). Proposed interpretation of fragmentation of RBC (C).

RBC peak elutes at 36.5 min in fragment ion chromatogram (Fig. 4A). Dissociation of RBC ion at *m*/*z* 435 inside the collision cell produces three characteristic and qualitative fragment ions at *m*/*z* 367, *m*/*z* 322, *m*/*z* 294 (Fig. 4B) that represented the loss of cyclopentyl ring, dimethyl amine and dimethyl carboxamide groups, respectively (Fig. 4C).

### 3.4. Identification of RBC related metabolites

After extraction of RBC RLMs incubations, fifteen μL were injected into mass spectrometer. RBC incubation revealed the characterization of nine *in vitro* metabolites and one cyano adduct, and the supposed pathways included oxidation, reduction, hydroxylation and cyanide addition (Table 3). All these metabolites were generated in all metabolic incubations when performed three time, this revealed the validity of the utilized method. These outcomes were approved by the absence of cyano conjugates and metabolites in different types of reaction controls.

**Table 3:** *In vitro* phase 1 metabolites and reactive intermediates of RBC

	Mass scan	Fragment ions	*R*_t_ (min)	Proposed phase 1 metabolic pathways
RBC	435	367, 322, 294	36.5	

***In vitro* phase I metabolism**				
M1	407	339, 322, 294	31.0	Double *N*-demethylation
M2	437	369, 324, 296	36.4	Reduction
M3	367	322, 294, 254	34.5	*N*-dealkylation of cyclopentyl ring
M4	451	383, 338, 310	43.5	Hydroxylation
M5	449	378, 336, 310, 265	38.9	Oxidation
M6	453	435, 336, 210, 100, 83	28.49	Hydroxylation and reduction
M7	453	435, 336, 210, 100, 83	34.37	Hydroxylation and reduction
M8	453	435, 336, 210, 100, 83	37.78	Hydroxylation and reduction
M9	453	385, 340 and *m*/*z* 311	43.46	Hydroxylation and reduction

**Cyano adducts**				
RBC476	476	449, 432, 378, 310, 265	44.7	Hydroxylation and cyanide addition (non-enzymatic reaction)

#### 3.4.1. Identification of M1

**Fig. 5:**
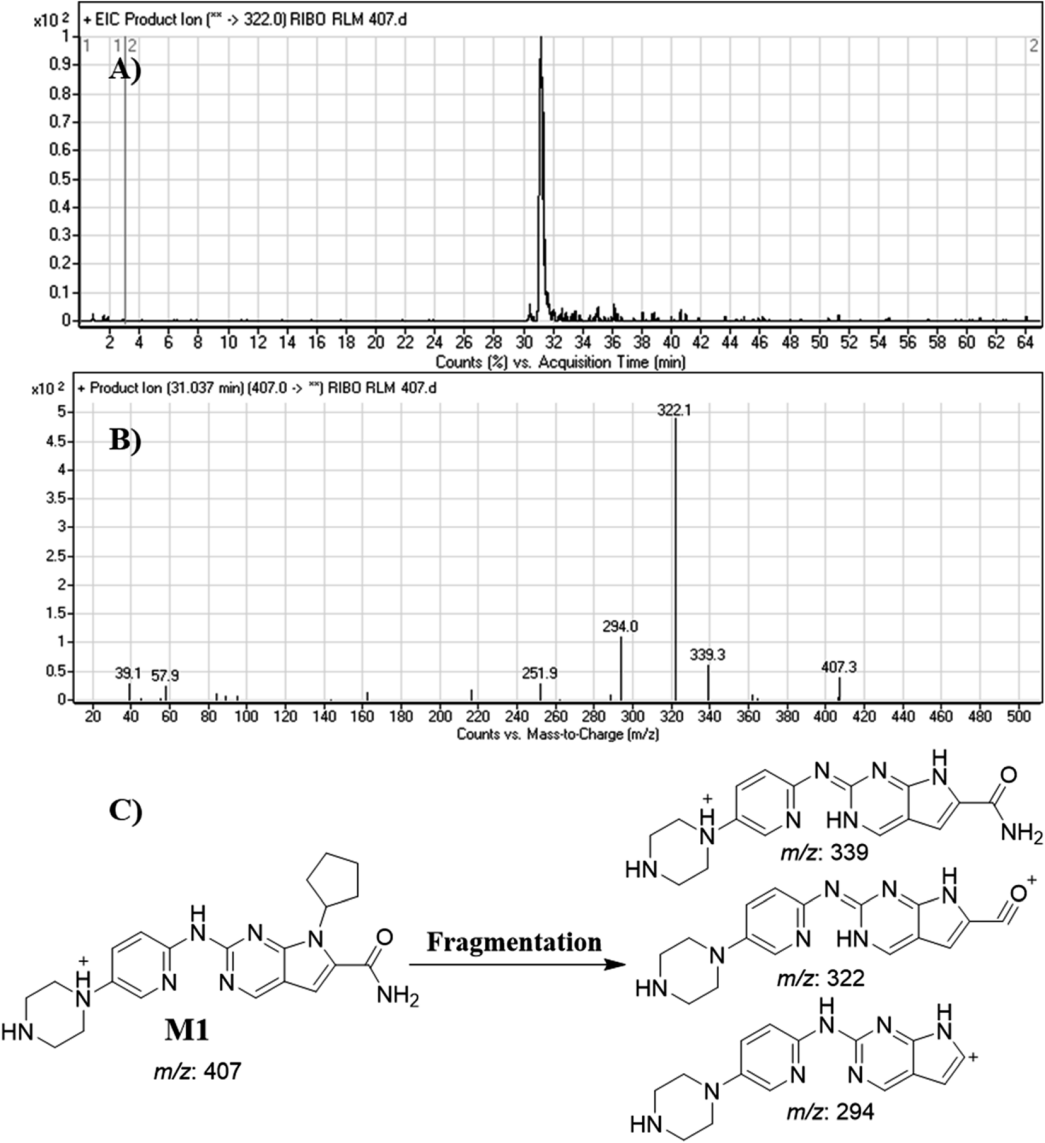
Product ion chromatogram of M1 (A). Product ion mass spectrum of M1 (B). Proposed interpretation of fragmentation of M1 (C).

M1 peak elutes at 31.0 min in fragment ion chromatogram (Fig. 5A). Dissociation of M1 ion at *m*/*z* 407 inside the collision cell generates three fragment ions at *m*/*z* 339, *m*/*z* 322 and *m*/*z* 294 (Fig. 5B). Comparing with fragment ions of RBC, a decrease of 28 *m*/*z* units (loss of 2 methyl groups) in one FI (*m*/*z* 339) was recognized that reveals a *N*-demethylation metabolic pathway at *N*,*N*-dimethyl carboxamide group that matched with the other FIs at *m*/*z* 322 and *m*/*z* 294 (Fig. 5C).

#### 3.4.2. Identification of M2

**Fig. 6:**
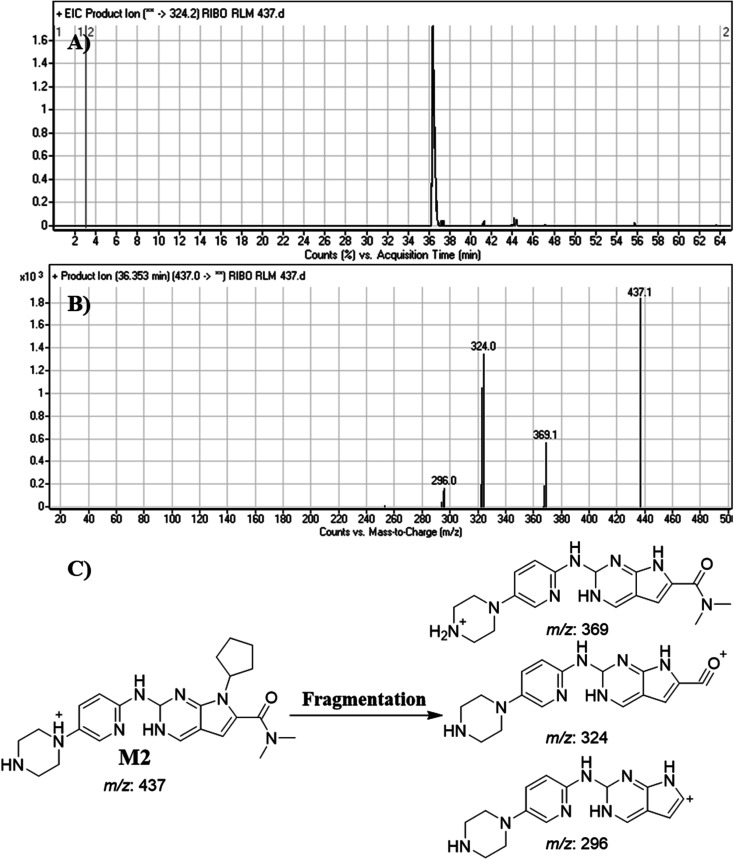
Product ion chromatogram of M2 (A). Product ion mass spectrum of M2 (B). Proposed interpretation of fragmentation of M2 (C).

M2 peak elutes at 36.35 min. in fragment ion chromatogram (Fig. 6A). Dissociation of M2 ion at *m*/*z* 437 inside the collision cell generates three fragment ions at *m*/*z* 369, *m*/*z* 324 and *m*/*z* 296 (Fig. 6B). Comparing with fragment ions of RBC, an increase of 2 *m*/*z* units in all the three FIs were recognized that reveals a reduction metabolic pathway at pyrimidine ring (Fig. 6C).

#### 3.4.3. Identification of M3

**Fig. 7:**
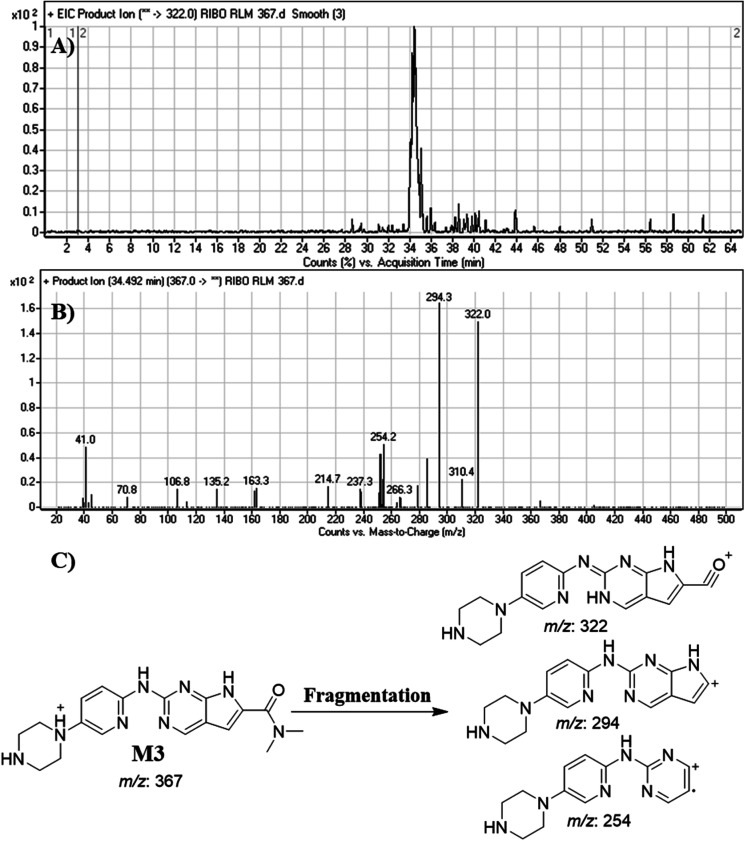
Product ion chromatogram of M3 (A). Product ion mass spectrum of M3 (B). Proposed interpretation of fragmentation of M3 (C).

M3 peak elutes at 34.5 min. in fragment ion chromatogram (Fig. 7A). Dissociation of M3 ion at *m*/*z* 367 inside the collision cell generates three fragment ions at *m*/*z* 322, *m*/*z* 294 and *m*/*z* 254 (Fig. 7B). Comparing with fragment ions of RBC, a decrease of 68 *m*/*z* units (loss of cyclopentyl group) was recognized that reveals a *N*-dealkylation metabolic pathway that matched with the FIs at *m*/*z* 322 and *m*/*z* 294 (Fig. 7C).

#### 3.4.4. Identification of M4

**Fig. 8:**
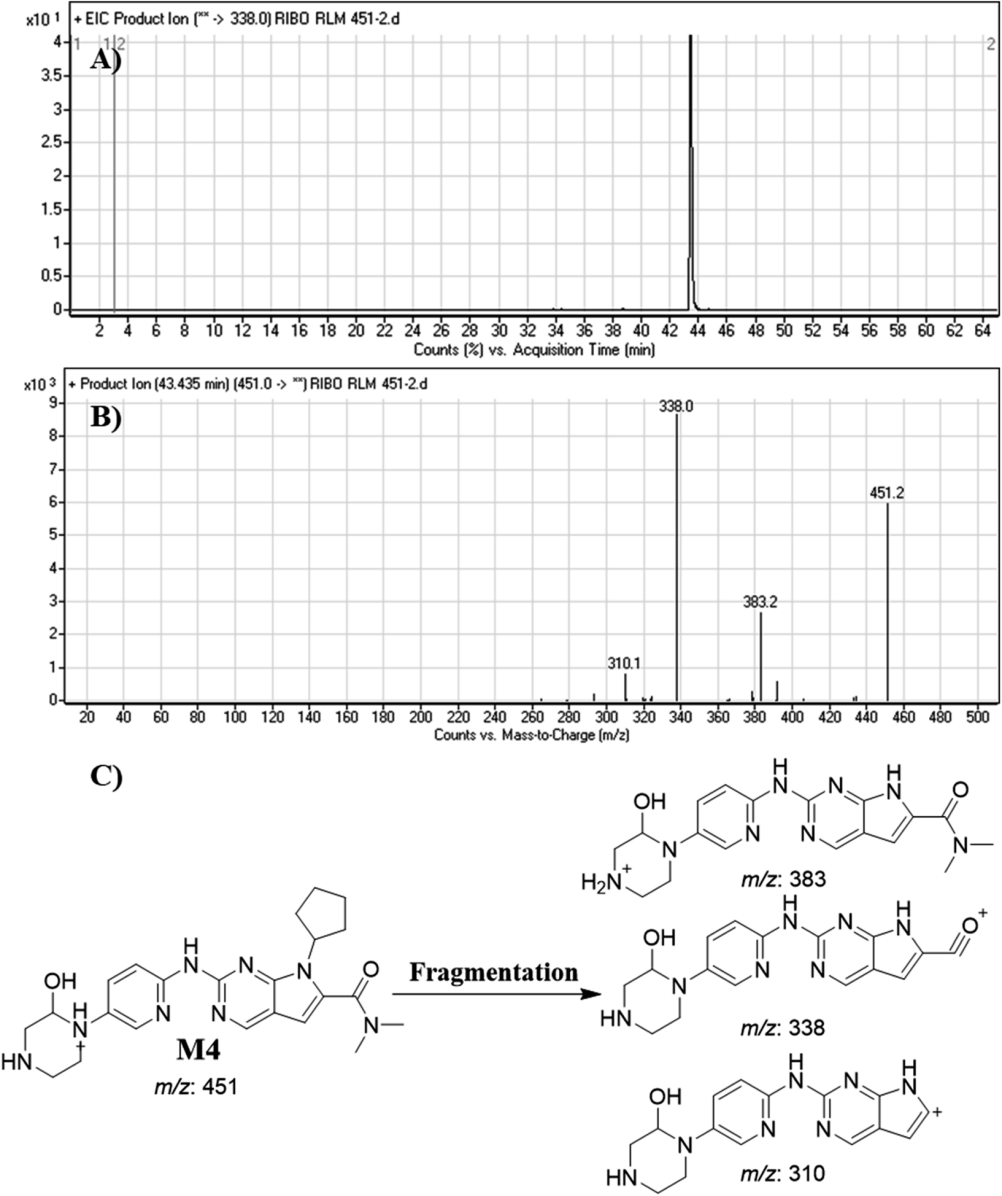
Product ion chromatogram of M4 (A). Product ion mass spectrum of M4 (B). Proposed interpretation of fragmentation of M4 (C).

M4 peak elutes at 43.44 min in fragment ion chromatogram (Fig. 8A). Dissociation of M4 ion at *m*/*z* 451 inside the collision cell generates three fragment ions at *m*/*z* 383, *m*/*z* 338 and *m*/*z* 310 (Fig. 8B). Comparing with fragment ions of RBC, an increase of 16 *m*/*z* units were recognized that reveals a hydroxylation metabolic pathway at piperazine ring (Fig. 8C).

#### 3.4.5. Identification of M5

**Fig. 9:**
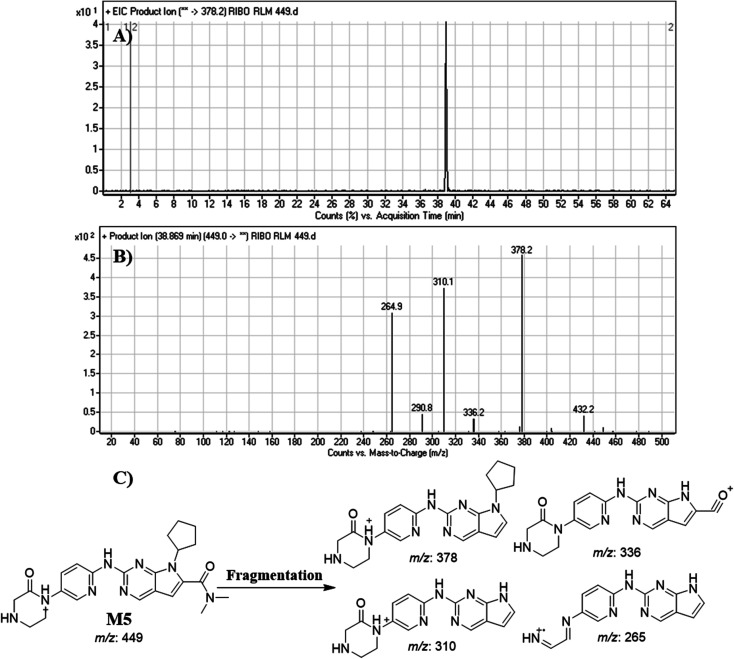
Product ion chromatogram of M5 (A). Product ion mass spectrum of M5 (B). Proposed interpretation of fragmentation of M5 (C).

M5 peak elutes at 38.87 min in fragment ion chromatogram (Fig. 9A). Dissociation of M5 ion at *m*/*z* 449 inside the collision cell generates three fragment ions at *m*/*z* 378, *m*/*z* 336, *m*/*z* 310 and *m*/*z* 265 (Fig. 9B). Comparing with fragment ions of RBC, an increase of 14 *m*/*z* units were recognized that reveals oxidation metabolic pathway at piperazine ring (Fig. 9C).

#### 3.4.6. Identification of M6, M7, M8 and M9

**Fig. 10:**
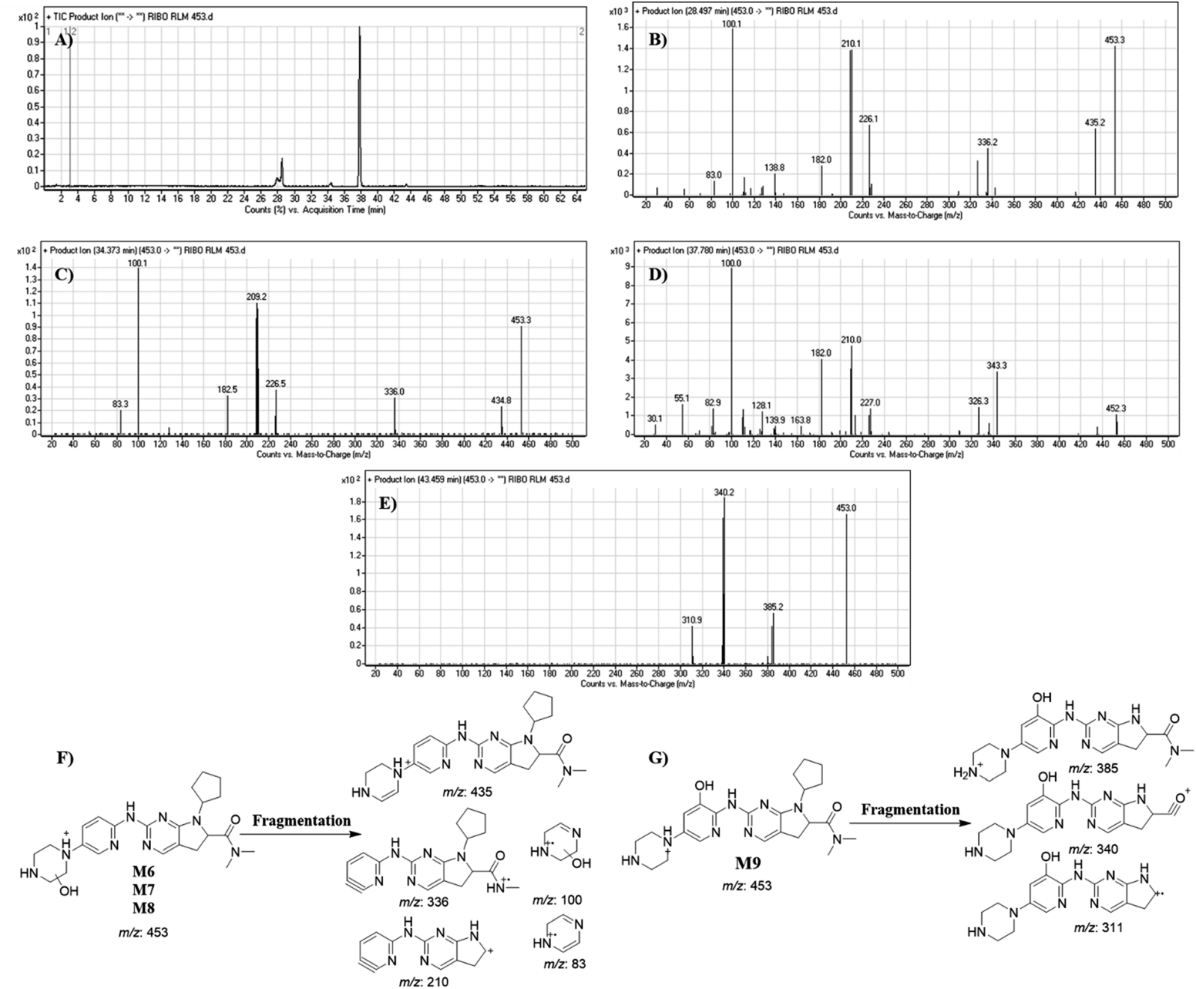
Product ion chromatogram of M6 to M9 (A). Product ion mass spectrum of M6 to M9 (B to E). Proposed interpretation of fragmentation of M6, M7 and M8 (F). Proposed interpretation of fragmentation of M9 (G).

RBC453 peaks appeared at different RTs: 28.49 min, 34.37 min, 37.78 and 43.46 min, respectively in fragment ion chromatogram (Fig. 10A) with different mass spectra. Dissociation of M6, M7 and M8 ions at *m*/*z* 453 inside the collision cell produces five fragment ions at *m*/*z* 435, *m*/*z* 336, *m*/*z* 210, *m*/*z* 100 and *m*/*z* 83 (Fig. 10B–D). Comparing with fragment ions of RBC, an increase of 18 *m*/*z* units were recognized that indicates hydroxylation metabolic pathway at piperazine ring and a reduction metabolic reaction at pyrimidine ring (Fig. 10F). The FI at *m*/*z* 435 revealed dehydration (loss of water) that verified hydroxylation at the piperazine ring at different positions generating three different metabolites.

Dissociation of M9 at *m*/*z* 453 inside the collision cell produces three fragment ions at *m*/*z* 385, *m*/*z* 340 and *m*/*z* 311 (Fig. 10E). Comparing with fragment ions of RBC, an increase of 18 *m*/*z* units were recognized that reveals hydroxylation metabolic reaction happened in piperazine ring and a reduction metabolic pathway at pyrimidine ring (Fig. 10G).

#### 3.4.7. Identification of RBC476 cyano adducts

**Fig. 11:**
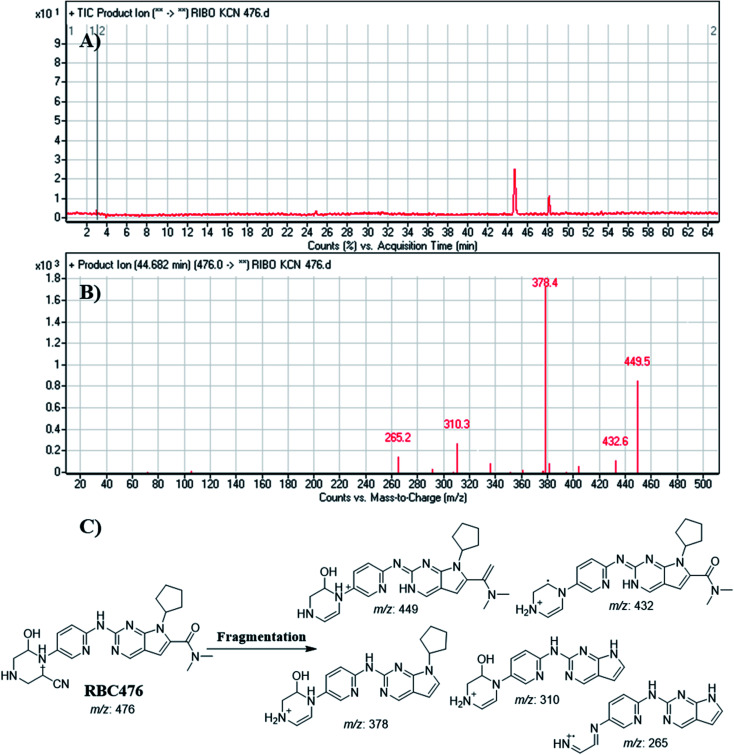
Product ion chromatogram of RBC476 (A). Product ion mass spectrum of RBC476 (B). Proposed interpretation of fragmentation of RBC476 (C).

RBC476 peak appeared at 40.68 min in fragment ion chromatogram (Fig. 11A). Dissociation of RBC476 ion at *m*/*z* 476 inside the collision cell produces five fragment ions at *m*/*z* 449 *m*/*z* 432, *m*/*z* 378, *m*/*z* 310 and *m*/*z* 265 (Fig. 11B). Comparing with fragment ions of RBC, fragment ion at *m*/*z* 449 shows a neutral loss of 27 (HCN) that is characteristic of cyano group addition. Fragment ion at *m*/*z* 449 reveals that all metabolic pathways occurred in piperazine ring. An increase of 25 *m*/*z* units reveals the cyano addition at piperazine ring (Fig. 11C).

### 3.5. Proposed bioactivation mechanism of RBC

**Fig. 12:**
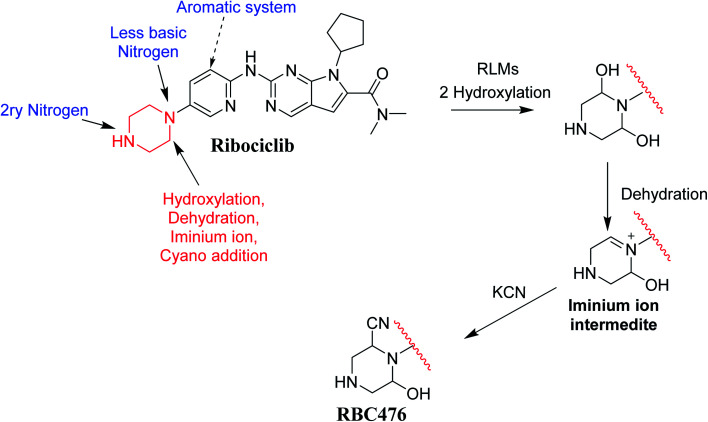
Proposed pathway for RBC476 generation and trapping strategy.

Fig. 12 shows the bioactivation pathway for RBC. The generation of RBC476 cyanide adduct revealed the generation of iminium unstable intermediates in piperazine moiety during *in vitro* metabolism of RBC. Metabolic hydroxylation pathway at piperazine moiety in RBC followed by loss of one water molecule (dehydration) lead to the formation of iminium ions intermediate which are reactive and unstable that can be captured by cyanide as nucleophile forming stable adduct that can be characterized in mass spectrometry. The generation mechanism of iminium intermediate formation of RBC is previously reported for cyclic tertiary amine containing drugs.^12,13,21,34^ Bioactivation of RBC generated one reactive iminium intermediate with a very low concentration, in part due to the involvement of only one basic nitrogen of the piperazine ring in the bioactivation sequence. One piperazine ring nitrogen is secondary and cannot go through bioactivation process, and the other nitrogen is attached to aromatic system that reduces its basicity (the lone pair on the nitrogen is partially delocalized into the pi system of the aromatic ring) and can go through bioactivation pathway when both α carbons undergo metabolic hydroxylation (Fig. 12). Side effects of RBC is expected to be lower comparing to abemaciclib.^12,36^

In summary, *in silico* tool has predicted the most vulnerable sites of metabolism (SOMs) in the structure of RBC. The suggested atomic sites for the metabolism in RBC structure were the same as seen in the generated metabolites in phase I metabolism. Additionally, the piperazine ring were less bioactivated as proposed by *in silico* experiments as only one reactive intermediate was formed.

### 3.6. *In silico* ADMET analysis for the proposed RBC derivatives

Discovery studio 4.5 was used to predict ADMET descriptors of RBC and its proposed derivatives as listed in Table 4. It was observed that low ability (value 3 = low) of the three molecules to penetrate BBB. So that they were expected to be safe toward CNS. Also, the selected molecules showed good human intestinal absorption. It is well known that many molecules have failed during clinical tests because of their absorption properties.^37^ The aqueous solubility logarithmic level of RBC, methyl-RBC and fluoro-RBC equal 3 indicating good aqueous solubility (value 0 = good). The cytochrome P450 2D6 model predicts the inhibitory and non-inhibitory behavior of 2D chemical structure. It was found that all the three molecules are non-inhibitors of CYP2D6 (0 = non inhibitor). So that, their liver dysfunction effect is not expected upon administration. Additionally, the three molecules are proposed to be non-hepatotoxic (the hepatotoxicity scores is one and hepatotoxicity probability values are less than 0.5). There are no differences in the ADMET properties of the methyl-RBC and fluoro-RBC from RBC.

**Table 4:** Predicted ADMET for the designed compounds and ribociclib

Comp.	BBB level	Solubility	Absorption level	PPB	CYP2D6	Hepatotoxicity probability	Hepatotoxicity
Ribociclib	3	3	0	0	0	0.119185	1
Methyl-RBC	3	3	0	0	0	0.119557	1
Fluoro-RBC	3	3	0	0	0	0.119344	1

### 3.7. Molecular docking for the proposed RBC derivatives

**Fig. 13:**
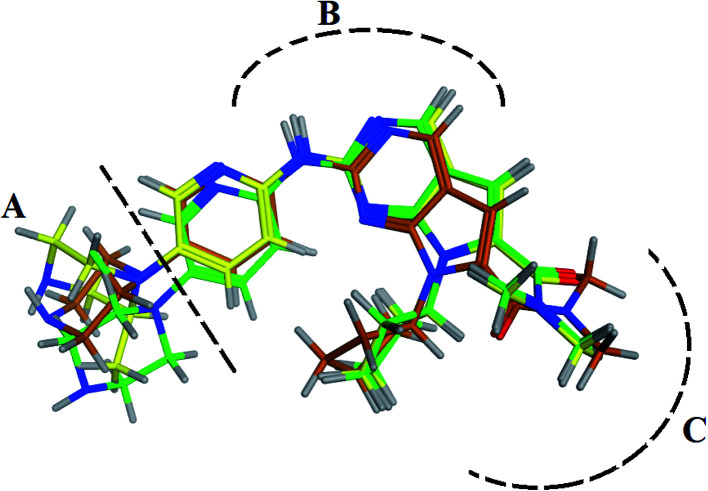
Superimposition of the crystallographic poses of molecules (green color: RBC, yellow: methyl-RBC, and brown color: fluoro-RBC) binding with CDK6 pocket, (A) solvent exposed region, (B) Hinge region and (C) hydrophobic back pocket.

RBC, methyl-RBC and fluoro-RBC forms two hydrogen bonds with Val101 of CDK6 *via* its 5*H*-pyrrolo[2,3-*d*]pyrimidin-2-amine moiety. RBC and methyl-RBC form hydrogen bond with Asp163 of CDK6 through its N, *N*-dimethyl acetamide while fluoro-RBC forms two hydrogen–π bond with Phe98 *via* its *N*,*N*-dimethyl acetamide. The hydrophobic packing of the piperazine group towards the solvent exposed region (Fig. 13) of the binding pocket of CDK6 (Table 5). The formed complexes reveal that the piperazine ring of the three molecules is stabilized by lying against a solvent-exposed ridge consisting of Asp104 and Thr107. For that, the addition of a methyl group in *meta* position for N-terminal of piperazine group increase the lipophilicity of the compound, and the hydrophobic effect are taken to refer to transfer of a molecule from a solvent-exposed to a more lipid-like environment such as interior of a protein pocket (Fig. 13).

**Table 5:** Binding mode of RBC and its proposed derivatives to CDK6 crystal structure (PDB:5L2T). Hydrogen bonds are shown in dashed lines

	2D	3D
RBC	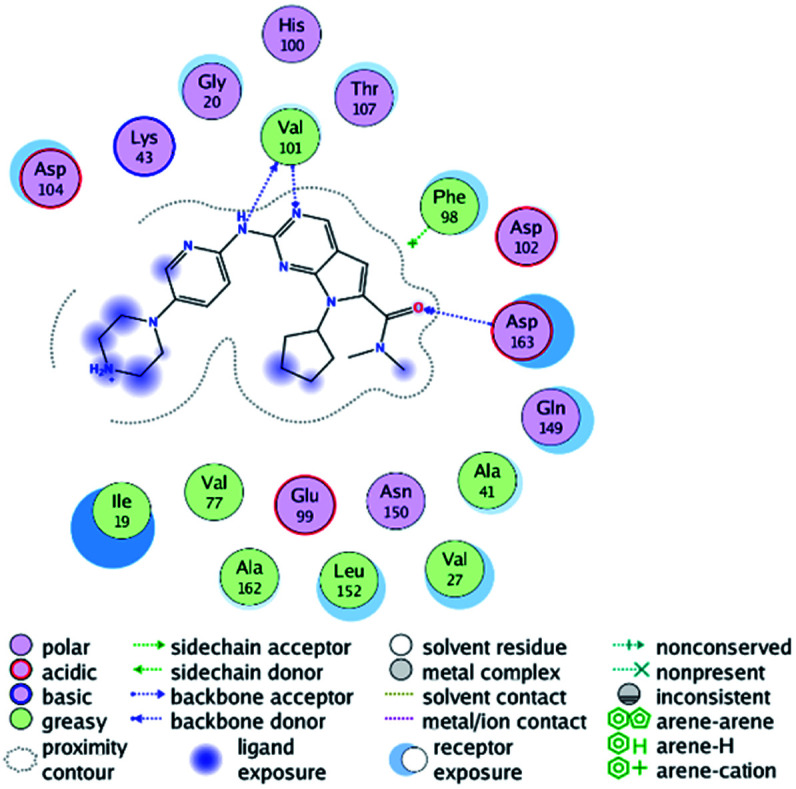	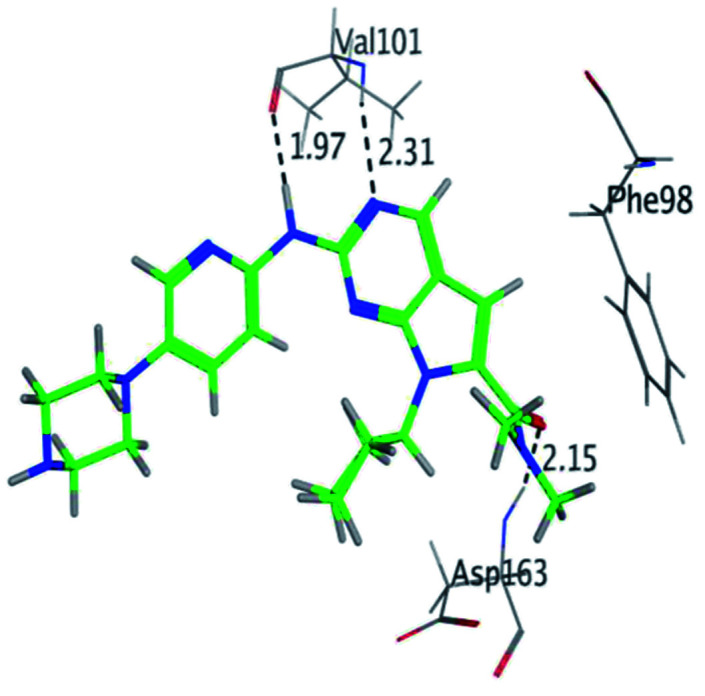
Methyl-RBC	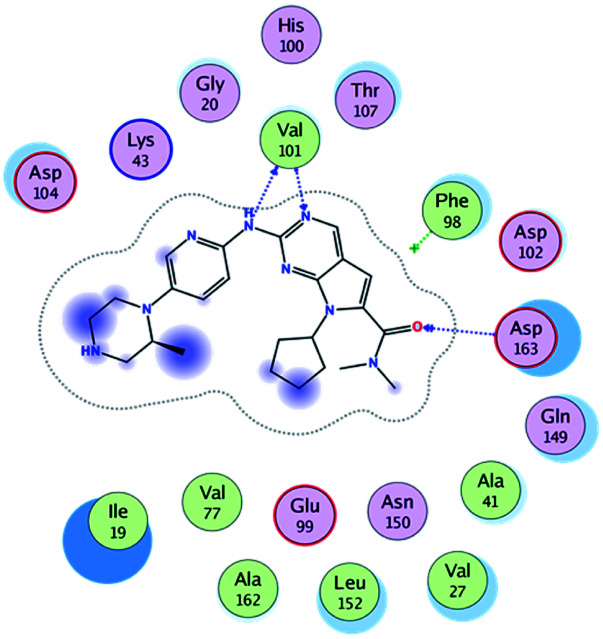	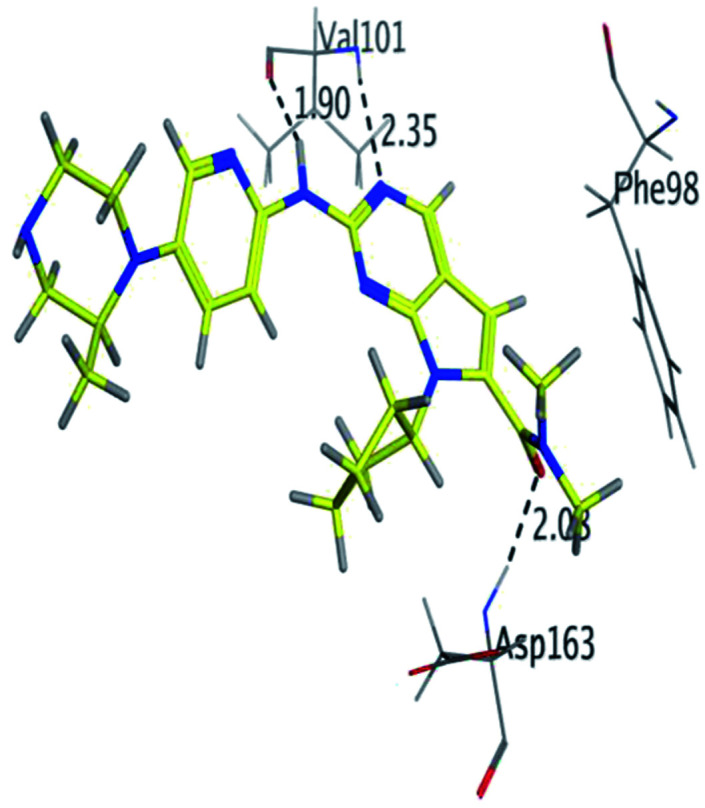
Fluoro-RBC	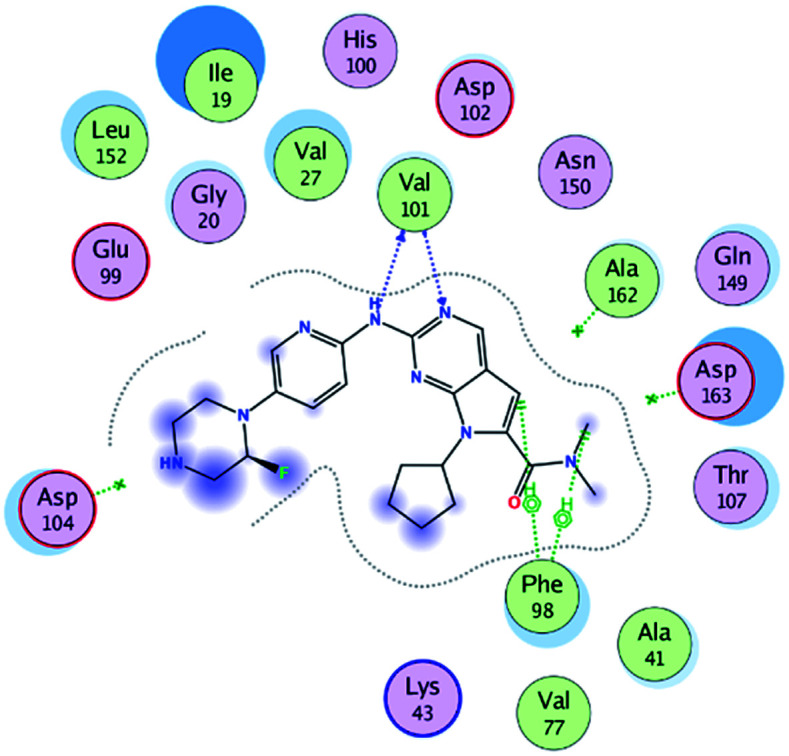	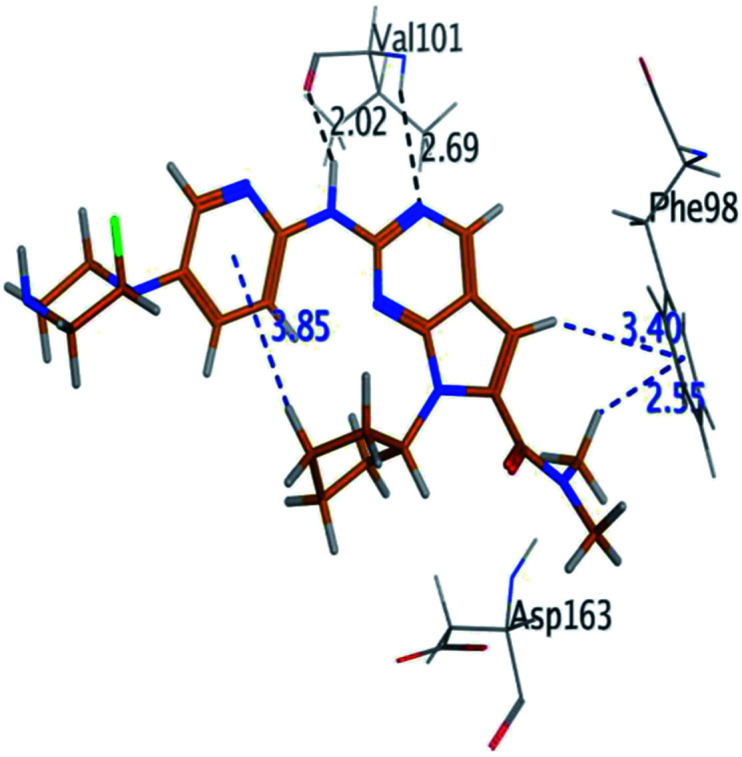

Where it has been observed that the hydrogen bond in the case of methyl-RBC (1.9 Å) is short when compared to RBC (1.97 Å) and fluoro-RBC (2.02 Å). This result explains that the piperazine group of methyl-RBC move to the binding pocket of the CDK6 or to a more lipid-like environment, while the piperazine group of fluoro-RBC is slightly transferred towards the solvent that occurred because of the effect of the fluoro substitution that affected the polarity of other groups of the compound which interact with the protein. This interpretation is consistent with the energy calculations, as the lowest energy predicted for the methyl-RBC (−9,278 kcal mol^−1^) compared to the highest expected fluoro-RBC formation energy (−8734 kcal mol^−1^) (Tables 5 and 6). The values of the scoring and refine RMSD (root mean square deviation) for good conformation of all ligand with CDK6 kinase receptor were recorded on (Table 6), in addition to the based selected conformation (2D and 3D shown in Table 5).

**Table 6:** Analysis of interaction between CDK6 and RBC and its proposed derivatives

Compounds	Ligand	Receptor	Interaction	Distance (Å)	Docking score (kcal mol^−1^)
RBC	H 57	O VAL 101	H-donor	1.97	−9.177
	N 55	HN VAL 101	H-acceptor	2.31	
	O 62	HN ASP 163	H-acceptor	2.15	
Methyl-RBC	H 56	O VAL 101	H-donor	1.90	−9.278
	N 54	HN VAL 101	H-acceptor	2.35	
	O 61	HN ASP 163	H-acceptor	2.08	
Fluoro-RBC	H 56	O VAL 101	H-donor	2.02	−8.734
	N 54	O VAL 101	H-acceptor	2.69	
	C 36	6-Ring PHE 98	H-pi	3.40	
	C 1	6-Ring PHE 98	H-pi	2.55	

From the outcomes, methyl-RBC and fluoro-RBC exhibited almost similar activity towards CDK6 if compared to RBC. This result is in accordance with the molecular docking outputs. Indeed, RBC had a lower binding affinity than the methyl-RBC and higher binding affinity than fluoro-RBC. This is due to the lower binding energy of both complexes (methyl-RBC and RBC) with CDK6 and the number of hydrogen bonds established in these complexes (Tables 5, 6 and Fig. 13). Three hydrogen bonds are formed between (RBC, methyl-RBC)- and CDK6 complexes, while in the case of fluoro-RBC with CDK6 complex, four hydrogen bonds are formed. Depending on the previous outcomes, methyl-RBC was predicted to have higher activity than that of the other two (RBC and fluoro-RBC).

## 4 Conclusions

**Fig. 14:**
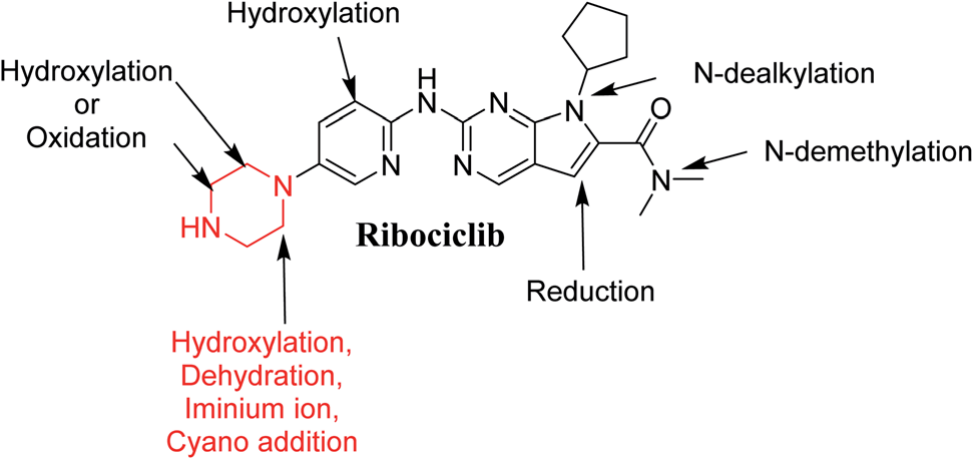
Proposed metabolic pathway for RBC and trapping strategy.

The current study involves *in silico*, *in vitro* (RLMs) and reactive (iminium intermediates) metabolite characterization of RBC using LC-MS/MS. The StarDrop WhichP450™ module efficiently identified vulnerable sites for metabolism. One potential reactive metabolite was detected and the bioactivation pathway was proposed and confirmed using DEREK software as a structural alert. Nine *in vitro* metabolites were identified (Fig. 14). Reactive intermediate generation mechanism of RBC may provide an explanation of its side effects. RBC exhibits less reactivity compared to abemaciclib due to less basicity of its piperazine ring nitrogen this could be a way for reducing reactive intermediates formation. Blocking or adding isosteric substituents to the available bioactive alpha position to the tertiary nitrogen of the piperazine ring moiety would likely interrupt or block metabolic hydroxylation that will stop the bioactivation sequence as confirmed using DEREK software. RBC and its proposed derivatives (methyl-RBC and fluoro-RBC) were analyzed for their ADMET properties that revealed there is no difference between RBC and its related derivatives. The methyl-RBC showed an increase of lipophilicity of piperazine ring pushing the molecule to inner of pocket for hydrophobic environment that increased the binding affinity of interaction. While, fluoro-RBC showed a decrease lipophilicity of piperazine ring which was one of reasons of decreasing the binding affinity of complexation. Methyl-RBC exhibited the best ADMET, docking analysis and less side effects compared to RBC and fluoro-RBC. Further drug discovery studies can be done depending on this concept allowing the development of new series of drugs with increased safety profile without affecting its pharmacological action.

## Ethics

Male Sprague-Dawley were maintained following the Animal Care Center instructions at King Saud University that were approved by Local Animal Care and Use Committee of KSU. The animal experimental procedure utilized in our current research was validated and approved by the King Saud University's Ethics Review Committee (number: KSU-SE-19-76).

## Authors' contributions

A. A. K., H. W. D., T. A. S and H. A. A. contributed in study design. T. A. S. and M. W. A. performed the data analysis, experimental work and aided drafting the manuscript. A. A. K., H. A. A. and H. W. D. directed the laboratory experimental work. H. W. D. is the corresponding author of this paper. The final draft manuscript was revised by all authors. A. H. B participated in preparing the revised version of the manuscript and performed the docking studies in the current manuscript. All authors approved the submission to the journal.

## Funding and acknowledgements

The authors would like to extend their sincere appreciation to the Deanship of Scientific Research at the King Saud University for funding this work through the Research Group Project No. RGP-322.

## Abbreviations

RBC: Ribociclib
CSL: Composite site liability
ACN: Acetonitrile
conc.: Concentration
RLM: Rat liver microsomes
SOM: Sites of metabolism
KCN: Potassium cyanide
TK: Tyrosine kinase
ESI: Electrospray ionizer
RRLC: Rapid resolution liquid chromatographer

## Conflicts of interest

The authors declare no competing interests.

## Supplementary Material

## References

[cit1] American cancer society,http://www.cancer.org/acs/groups/cid/documents/webcontent/003090-pdf.pdf

[cit2] McGuire A., Brown J. A., Malone C., McLaughlin R., Kerin M. J. (2015). Cancers.

[cit3] National Campaign for Breast Cancer Awareness, http://www.moh.gov.sa/en/HealthAwareness/Campaigns/Breastcancer/Pages/stat.aspx

[cit4] Takeuchi K., Ito F. (2011). Biol. Pharm. Bull..

[cit5] Traxler P. (2003). Expert Opin. Ther. Targets.

[cit6] Kwapisz D. (2017). Breast Canc. Res. Treat..

[cit7] Bilgin B., Sendur M. A. N., Şener Dede D., Akıncı M. B., Yalçın B. (2017). Curr. Med. Res. Opin..

[cit8] Shohdy K. S., Lasheen S., Kassem L., Abdel-Rahman O. (2017). Ther. Adv. Drug Saf..

[cit9] Kadi A. A., Darwish H. W., Abuelizz H. A., Alsubi T. A., Attwa M. W. (2019). R. Soc. Open Sci..

[cit10] Kadi A. A., Amer S. M., Darwish H. W., Attwa M. W. (2017). RSC Adv..

[cit11] Kadi A. A., Attwa M. W., Darwish H. W. (2018). RSC Adv..

[cit12] Attwa M. W., Kadi A. A., Alrabiah H., Darwish H. W. (2018). J. Pharm. Biomed. Anal..

[cit13] Attwa M. W., Kadi A. A., Darwish H. W., Alrabiah H. (2018). Clin. Chim. Acta.

[cit14] Attwa M. W., Kadi A. A., Darwish H. W., Amer S. M., Al-shakliah N. S. (2018). Chem. Cent. J..

[cit15] Amer S. M., Kadi A. A., Darwish H. W., Attwa M. W. (2017). Chem. Cent. J..

[cit16] Knowles S. R., Uetrecht J., Shear N. H. (2000). Lancet.

[cit17] Ju C., Uetrecht J. (2002). Curr. Drug Metab..

[cit18] Ma S., Zhu M. (2009). Chem.-Biol. Interact..

[cit19] Stepan A. F., Walker D. P., Bauman J., Price D. A., Baillie T. A., Kalgutkar A. S., Aleo M. D. (2011). Chem. Res. Toxicol..

[cit20] Attwa M. W., Kadi A. A., Darwish H. W., Amer S. M., Al-Shakliah N. S. (2018). Chem. Cent. J..

[cit21] Kadi A. A., Darwish H. W., Attwa M. W., Amer S. M. (2016). RSC Adv..

[cit22] Attwa M. W., Kadi A. A., Abdelhameed A. S. (2019). J. Pharm. Biomed. Anal..

[cit23] Chavan B. B., Tiwari S., Shankar G., Nimbalkar R. D., Garg P., Srinivas R. B., Talluri M. V. N. K. (2018). J. Pharm. Biomed. Anal..

[cit24] Chen P., Lee N. V., Hu W., Xu M., Ferre R. A., Lam H., Bergqvist S., Solowiej J., Diehl W., He Y.-A. (2016). Mol. Canc. Therapeut..

[cit25] von Jagow R., Kampffmeyer H., Kiese M. (1965). N. Schmied. Arch. Pharmacol..

[cit26] Kadi A. A., Attwa M., Darwish H. W. (2018). RSC Adv..

[cit27] Attwa M. W., Kadi A. A., Alrabiah H., Darwish H. W. (2018). J. Pharm. Biomed. Anal..

[cit28] Attwa M. W., Kadi A. A., Abdelhameed A. S. (2020). J. Sep. Sci..

[cit29] Attwa M. W., Kadi A. A., Darwish H. W., Amer S. M., Alrabiah H. (2018). Eur. J. Mass Spectrom..

[cit30] Hughes T. B., Dang N. L., Miller G. P., Swamidass S. J. (2016). ACS Cent. Sci..

[cit31] Matlock M. K., Hughes T. B., Swamidass S. J. (2015). Bioinformatics.

[cit32] Zaretzki J., Matlock M., Swamidass S. J. (2013). J. Chem. Inf. Model..

[cit33] Waterborg J. H., Matthews H. R. (1994). Methods Mol. Biol..

[cit34] Amer S. M., Kadi A. A., Darwish H. W., Attwa M. W. (2017). RSC Adv..

[cit35] Chen P., Lee N. V., Hu W., Xu M., Ferre R. A., Lam H., Bergqvist S., Solowiej J., Diehl W., He Y. A., Yu X., Nagata A., VanArsdale T., Murray B. W. (2016). Mol. Cancer Ther..

[cit36] Eggersmann T. K., Degenhardt T., Gluz O., Wuerstlein R., Harbeck N. (2019). BioDrugs.

[cit37] El-Gamal K. M., El-Morsy A. M., Saad A. M., Eissa I. H., Alswah M. (2018). J. Mol. Struct..

