# Correction: *In silico* and *in vitro* metabolism of ribociclib: a mass spectrometric approach to bioactivation pathway elucidation and metabolite profiling

**DOI:** 10.1039/d0ra90070b

**Published:** 2020-06-23

**Authors:** Thamer A. Alsubi, Mohamed W. Attwa, Ahmed H. Bakheit, Hany W. Darwish, Hatem A. Abuelizz, Adnan A. Kadi

**Affiliations:** Department of Pharmaceutical Chemistry, College of Pharmacy, King Saud University P. O. Box 2457 Riyadh 11451 Saudi Arabia hdarwish@ksu.edu.sa +966 1146 76 220 +966 1146 70237; Students’ University Hospital, Mansoura University Mansoura 35516 Egypt; Analytical Chemistry Department, Faculty of Pharmacy, Cairo University Kasr El-Aini St. Cairo 11562 Egypt

## Abstract

Correction for ‘*In silico* and *in vitro* metabolism of ribociclib: a mass spectrometric approach to bioactivation pathway elucidation and metabolite profiling’ by Thamer A. Alsubi *et al.*, *RSC Adv.*, 2020, **10**, 22668–22683, DOI: 10.1039/D0RA01624A.

The authors regret that the email address for the corresponding author was shown incorrectly in the original article. The corrected email address is as shown above.

The Royal Society of Chemistry apologises for these errors and any consequent inconvenience to authors and readers.

## Supplementary Material

